# Acute kidney injury in critically ill cancer patients: an update

**DOI:** 10.1186/s13054-016-1382-6

**Published:** 2016-08-02

**Authors:** Norbert Lameire, Raymond Vanholder, Wim Van Biesen, Dominique Benoit

**Affiliations:** 1 Renal Division, Department of Medicine, University Hospital, 185 De Pintelaan, 9000 Gent, Belgium; 2 Medical Intensive Care Unit, University Hospital, 185 De Pintelaan, 9000 Gent, Belgium

## Abstract

Patients with cancer represent a growing group among actual ICU admissions (up to 20 %). Due to their increased susceptibility to infectious and noninfectious complications related to the underlying cancer itself or its treatment, these patients frequently develop acute kidney injury (AKI). A wide variety of definitions for AKI are still used in the cancer literature, despite existing guidelines on definitions and staging of AKI. Alternative diagnostic investigations such as Cystatin C and urinary biomarkers are discussed briefly. This review summarizes the literature between 2010 and 2015 on epidemiology and prognosis of AKI in this population. Overall, the causes of AKI in the setting of malignancy are similar to those in other clinical settings, including preexisting chronic kidney disease. In addition, nephrotoxicity induced by the anticancer treatments including the more recently introduced targeted therapies is increasingly observed. However, data are sometimes difficult to interpret because they are often presented from the oncological rather than from the nephrological point of view. Because the development of the acute tumor lysis syndrome is one of the major causes of AKI in patients with a high tumor burden or a high cell turnover, the diagnosis, risk factors, and preventive measures of the syndrome will be discussed. Finally, we will briefly discuss renal replacement therapy modalities and the emergence of chronic kidney disease in the growing subgroup of critically ill post-AKI survivors.

## Background

The association between cancer and kidney disease has long been recognized but received extra attention after the creation of a ‘new’ nephrological subspecialty, called ‘onco-nephrology’ [1].

Over the past several years, important advances have occurred in the treatment and supportive care of patients with malignancies. Given the increasing number of novel therapeutics and the complexity of cancer treatment protocols, nephrologists and intensivists face the difficult task to stay updated on the advances in onco-nephrology.

Acute kidney injury (AKI) and electrolyte disturbances are the most common forms of renal disease that occur in a patient with cancer [2]. As in practically all other causes of AKI, preexisting chronic kidney disease is one of the most important predisposing risk factors, next to extracellular volume depletion due to vomiting or diarrhea, urinary tract obstruction, fluid and electrolyte disturbances, exposure to contrast media, nephrotoxic antibiotics, nonsteroidal anti-inflammatory drugs, and nephrotoxicity induced by some of the anticancer treatments [2]. During recent years, a great number of so-called targeted therapies have been introduced, but unfortunately many of these drugs have renal side effects, AKI being one of them [3, 4].

Cancer patients are particularly at risk for AKI secondary to infection and sepsis [5, 6], tumor lysis syndrome (TLS) [7], kidney damage induced by immunosuppression after hematopoietic stem cell transplantation (HSCT) [8], and direct effects from the primary malignancy [9].

The development of AKI can jeopardize further cancer treatment, increase the toxicity and/or reduce the delivery of chemotherapy, and exclude patients from clinical trials.

The incidence and severity of AKI varies depending on the type and stage of cancer, treatment course, and comorbidities [10–12]. As in other clinical settings, AKI morbidity and mortality increase in the presence of critical illness or the need for renal replacement therapy (RRT) [10].

Most studies of AKI in cancer patients focus on the relation between hemato-oncological malignancies and the kidney [13], especially in specific conditions such as multiple myeloma (MM) [14] and HSCT [15]. However, only few studies focused specifically on the vast group of nonhematologic critically ill cancer patients with AKI [16].

This narrative review aims to provide an update on the relation between AKI and the critically ill cancer patient. Literature from the last 5 years (between 2010 and 2015) is covered and the article represents a follow-up of a previously published review on this topic [10].

## Diagnosis of AKI in cancer patients

Because the incidence of AKI depends on its definition, comparisons are most reliable if based on studies using a more or less uniform definition, as in the Risk, Injury, Failure, Loss, End stage renal disease (RIFLE), Acute Kidney Injury Network (AKIN), and Kidney Diseases Improving Global Outcomes (KDIGO) classifications (for review see [17]). The KDIGO criteria stage patients according to changes in serum creatinine (SCr) and urine output (UO) rather than changes in glomerular filtration rates (GFR). Both SCr and UO criteria are important predictors, and use of the KDIGO definition without assessment of UO underestimates the incidence and grade of AKI and can delay diagnosis [18].

Patients with cancer often have decreased creatinine production secondary to loss of cell mass, low protein intake, cachexia, inflammation, volume expansion, or medications. All these affect SCr independent of renal function, and thus limit the sensitivity of creatinine as a marker of kidney injury. Therefore, a fixed (arbitrary) level of SCr as a marker of AKI (i.e., >1.5 or 2.0 mg/dl) is inadequate as affected patients may be overlooked.

## Cystatin C

Cystatin C (CysC) is a 13-kDa cystein protease inhibitor produced in all nucleated cells at a relatively constant level and is filtered freely by glomeruli and is neither secreted nor reabsorbed by renal tubules but undergoes almost complete catabolism by proximal tubular cells. With a half-life of about 2 hours, serum CysC reflects GFR better than creatinine. CysC is relatively stable, and can be accurately measured with automatic analyzers, although at manifold the cost of SCr.

In a meta-analysis of 13 studies across different settings, including ICU patients, the likelihood ratio for serum CysC level to predict AKI was 23.5 (95 % CI, 14.2–38.9), with sensitivity and specificity of 0.84 and 0.82, respectively. The area under the receiver operating characteristic curve (AUROC) of serum CysC level to predict AKI was 0.96 (95 % CI, 0.95–0.97). Subgroup analysis showed that serum CysC was of diagnostic value when measured early (within 24 hours after renal insult or ICU admission) [19].

In the past, the value and usefulness of CysC in cancer patients were doubtful, because concentrations were thought to be influenced by the extent of malignancy. More recent studies have now found no correlation between serum CysC concentration and tumor burden [20].

It is important to note that the diagnostic and prognostic characteristics of CysC for AKI remain incompletely defined; not only anthropometric parameters (age, obesity), but current cigarette smoking, CRP levels, use of corticosteroids, and changes in thyroid function are associated with higher serum CysC levels, independent from GFR. This suggests that serum CysC is a marker of inflammation as well as of renal function [21].

## Novel biomarkers

Because traditional markers like SCr, UO, and CysC are considered insensitive markers of renal injury, extensive efforts were spent over the last 15 years to identify and validate novel biomarkers that are more sensitive for onset of injury, specific for prediction, and with greater discrimination for injury severity (for recent in-depth reviews see [22]).

Essentially three types of novel biomarkers in the field of AKI have been developed; the first group are inflammatory biomarkers, including neutrophil gelatinase-associated lipocalin (NGAL) and proinflammatory cytokines, like IL-6 and IL-18; the second group includes cell injury biomarkers such as kidney injury molecule-1 (KIM-1), liver fatty acid binding protein (L-FABP), sodium/hydrogen exchanger 3 (NHE-3), and netrin 1; and the third group has recently been developed and consists of cell cycle markers, like urinary tissue inhibitor of metalloproteinases-2 (TIMP-2) and insulin like growth factor-binding protein 7 (IGFBP-7).

Probably the most promising application of these biomarkers in oncology is their potential for early detection of nephrotoxicity by antineoplastic agents during their development phase and in preclinical studies. Some studies [23, 24] have demonstrated that the presence of antineoplastic nephrotoxicity was associated with an increase in AKI biomarkers.

Two commercially available AKI biomarkers NGAL and [TIMP-2]•[IGFBP7] can be measured on standard platforms used for routine biochemistry or with a point-of-care device.

Recent findings, however, challenge the robustness and utility of cell cycle arrest biomarkers for the prediction of AKI in general ICU patients with heterogeneous diagnoses, differing comorbidities, and multiple sources of inflammation [25]. In particular, the association with inflammation may obscure the picture in cancer patients. There are at present no studies that have evaluated whether earlier diagnosis of AKI by these biomarkers indeed results in better patient outcomes, and consequently in decreased costs [26].

## Patients with cancer and AKI

A substantial rise in AKI has been noted worldwide [27] and in the subgroup of noncritically ill and critically ill patients with cancer [28, 29].

Cancer patients represent about 20 % of ICU admissions [30, 31]. In a recent multinational study, an even distribution in cancer patients was observed between the subgroup without (18.8 %) vs with (20.5 %) AKI [30]. As in many other AKI populations, even small increases of SCr (0.2 mg/dl–17.6 μmol/l), previously considered trivial, are associated with prolonged ICU stay and increased mortality [32], and this is independent from severity of illness or other risk factors.

In the Dutch National Intensive Care Evaluation (NICE) database [33] AKI occurred in 19.4 % of hematological patients admitted to the ICU, which is comparable with incidence rates in patients with chronic liver cirrhosis and chronic heart failure, but higher than in patients with solid tumors (11 %).

## Overall prognosis of critically ill cancer patients

The overall prognosis of patients with cancer is strongly dependent on the type of ICU admission and underlying cancer. In a cohort including 1257 patients with cancer, in those admitted to the ICU (404, 32.1 %) overall ICU and hospital mortalities were 20.8 % and 28.6 %, respectively [34]. As expected, hospital mortality was significantly higher for medical (69.4 %) and emergency surgical patients (49.3 %) than for scheduled surgical patients (5.7 %; *p* < 0.001). After exclusion of scheduled surgical patients, those with solid tumors had a lower mortality compared with those with hematological malignancies (56.0 % and 67.2 %, *p* = 0.030).

Of the 3147 patients enrolled in the SOAP study, 473 (15 %) had a malignancy, 404 (85 %) solid cancer, and 69 (15 %) hematological cancer [35]. Patients with solid cancers had a similar severity of illness compared with the noncancer population, but cancer patients were older and were more likely to be admitted after surgery. Patients with hematological malignancies were the most severely ill and more commonly suffered from sepsis, acute lung injury/acute respiratory distress syndrome, and renal failure than patients with other malignancies; these patients also had the highest hospital mortality rate (58 %). The outcome of all cancer patients was comparable with that of the noncancer population, with a 27 % hospital mortality rate. However, in the subset of patients with more than three failing organs, greater than 75 % of patients with cancer died compared with about 50 % of patients without cancer (*p* = 0.01).

Similar results were obtained in the Dutch NICE database [33]. ICU (33.8 %) and hospital (47.4 %) mortalities were higher in hematological compared with nonhematological patients (17.9 % and 26.3 %, respectively). Important, however, is that 60-day mortality in patients with hematological malignancies and solid cancer was similar to patients with other classical severe comorbidities, and in the ranges of those for critically ill hematological patients in ICUs in France and Belgium [36].

Overall mortality has sharply dropped among ICU patients with hematological malignancies, including those requiring mechanical ventilation [37]. However, whether this improvement is due to better ICU triage or to real improvements in supportive therapy is unclear [38].

## Epidemiology and prognosis of AKI in critically ill cancer patients

AKI requiring RRT is more common in ICU patients with vs without cancer [9]. Hospital mortality rates are high in cancer patients with AKI, especially when RRT is required [9, 16].

The multiple types of renal injury that may precede or are concurrent with critical illness make cancer patients particularly vulnerable to the development of AKI, which therefore frequently occurs in the setting of multiple organ dysfunction.

Table 1 summarizes studies over the last 5 years on epidemiology of AKI in adult critically ill cancer patients admitted to the ICU. The table contains data on the type of study (monocentric or multicentric), type of cancer (hemato-oncology or solid tumors), reasons for admission to the ICU, applied definition of AKI, calculated incidence of AKI, causes or contributing factors to the AKI, survival data, indications and type of RRT, and where possible some long-term effects of AKI on patient health.

**Table 1:** Summary of publications between 2010 and 2015 on critically ill cancer patients with AKI

Reference	Multicenter (Yes/No)	Type of cancer	Population	Reasons for admission to ICU	Definition of AKI	Incidence of AKI	Cause(s) of AKI/contributing factors	Survival/mortality of AKI cancer patients (%)	Survival/mortality of non-AKI cancer patients (%)	Indications for RRT	RRT modality	Long-term prognosis for AKI patients
[46]	No	Hemato	537 with induction chemo	-	50 % rise in SCr above baseline RIFLE	36 % RIFLE R: 15 % I: 10 % F: 11 %	?	Mortality RIFLE R: 13.6 % I: 19.6 % F: 61.7 %	Mortality: 4 %	Indications? RRT: 8 %	?	
[48]	No	Hemato	344	Infections/noninfectious complications	?	16.6 %	?	?	?	?		
[47]	No	Hemato	94	Severe sepsis/shock: 71 % Renal failure: 17 % Respiratory failure: 12 %	Need for RRT RIFLE	RIFLE R:7 % I: 33 % F: 60 %	Septic AKI: 54 % Nonseptic AKI: 46 % Nephrotoxic drugs: 15/94 Hypovolemic: 13/94 TLS: 11/94	23.5 % survival	-		CVVHDF/CVVHF	23 % of survivors persistent AKI after ICU discharge
[32]	No	All	3795 medical/surgical pts	Oncology ICU Not specified	Assess link to outcome of increases of SCr cfr RIFLE	21.8 % RIFLE R: 12.5 % I: 4.9 % F: 4.5 %	Multiple	Creat rise: 10 %: Mortality: 15–25 %; Creat rise 25 %:	?	? RRT: 19 % in nonsurvivors, 1.6 % in survivors	?	?
								Mortality: 14–30 % Rise in SCr of 25 % (≥48 h) Mortality: 36 %	No rise in SCr of 25 % for ≥ 48 h: Mortality: 15 %			
[40]	Yes	All	All AKI 773 pts Ca pts: 118 (15.2 %) Solid cancer: 73 % Hemato: 27 %	Serious comorbidities	RIFLE before start of RRT	118/773 100 % AKI RIFLE R: 25 % I: 24 % F: 52 %	Sepsis: 78 % Ischemia/shock: 74 % Toxins/contrast: 32 %	Ca pts hospital survival: 12 % of all survivors Non Ca pts hospital survival: 7 % of all survivors Overall survival: 30 %	-	Azotemia: 58 % Fluid overload: 55 % Acidosis: 48 %	CRRT: 89 %	?
[45]	No	Hemato	199 pts onco	Oncology ICU Respiratory failure: 33 % Postoperative: 20 % Sepsis: 21.1 %	? Need for RRT?	8 % on ICU admission	?	RRT survivors: 38 % RRT nonsurvivors: 62 %	?	79 (40.9 %)	?	?
[44]	No	All	477 ca pts	ICU surgical pts	AKIN criteria	10.3 % on ICU admission	Post surgery	Hospital mortality: 13 % ICU mortality: 10.9 %	Hosp mortality: 1.5 % ICU mortality: 1 %	?	?	?
[6]	No	All	563 ca pts with sepsis Solid cancer: 77 % Hemato: 23 %	Sepsis	? Need for RRT?	20 % on RRT	Sepsis	? Overall mortality: ICU: 51 % Hospital: 65 % 6 month: 72 %	?	?	?	?
[11]	No	Hemato	200 pts	Start chemo 1 day before ICU See [73]	RIFLE	Total:68.5 % RIFLE R: 27 % I: 19.7 % F: 53.3 %	Prerenal: 48.2 % TLS: 43.8 % ATN: 28.5 % Nephrotoxicity: 20.4 % Multiple causes: 45.3 %	ICU mortality: 35 % Hospital mortality: 35 %	ICU mortality: 7.9 % Hospital mortality: 19.1 %	? 52.7 % RRT (during ICU)	IHD: 63.8 % CRRT: 19.4 % Both: 16.6 %	Complete hemato remission At 6 months AKI: 39.4 % Non AKI: 68.3 %
[42]	No	All	162 ca pts Solid cancer: 104 Hemato: 35.8 %	Septic shock: 66.7 % Respiratory failure: 63.6 %	? Need for RRT?	AKI: 30 % Hemato: 41 % Solid cancer: 23 %	?	Mortality AKI:26 %		Total RRT: 33 % Hemato: 38 % Solid cancer: 30 %	?	?
[43]	No	All	56 pts with chemo	Chemo	?	16 % Hospital nonsurvivors: 13 % Hospital survivors: 18 %	?	?		39 %	CVVHF	?
[33]	Yes	All	Hemato: 1741 Solid tumors: 602	Not specified	?	Hemato: 20 % Solid cancer: 11 %	?	?	?			
[30]	Yes	All	Ca pts: 357 (19.8 % of all admissions) AKI-EPI study	Multiple	KDIGO	59 %	?	?	?	?	?	?
[41]	Yes	Hemato	Neutropenic pts: 289	Sepsis: 80 % Acute respiratory failure: 64 % Shock: 58 %	? Need for RRT?	18 %	?	19.85 % mortality	?	?	?	?
[39]	Yes	Hemato	Hemato: 1009	Acute respiratory failure: 62.4 % Shock: 42.4 % AKI: 30.5 % Coma: 22.3 % Urgent chemo: 6.9 %	AKIN	During ICU stay: 66.5 % AKIN1: 38.4 % AKIN2: 11.2 % AKIN3: 50.4 %	Nephrotoxic agents:25.3 % Sepsis/shock: 31.3 %	Hospital mortality: 44.3 % AKIN stage dependent	Hospital mortality: 25.4 %	RRT: 271 (26.9 %) RRT-AKIN AKIN1: 19.6 % AKIN2: 9.9 % AKIN3: 64.2 %	CRRT: 136 pts IHD: 135 pts	12.9 % of survivors RRT dependent

*AKI* acute kidney injury, *AKIN* Acute Kidney Injury Network, *ca pts* cancer patients, *chemo* chemotherapy, *CRRT*, continuous renal replacement therapy, *CVVHDF* continuous venovenous hemodiafiltration, *CVVHF* continuous venovenous hemofiltration, *hemato* hematology, *IHD* intermittent hemodialysis, *KDIGO* and Kidney Diseases Improving Global Outcomes, *onco* oncology, *pts* patients, *RIFLE* Risk, Injury, Failure, Loss, End stage renal disease, *RRT* renal replacement therapy, *SCr* serum creatinine, *TLS* tumor lysis syndrome, *ATN* acute tubular necrosis

The table contains 15 publications of which only five are multicentric [30, 33, 39–41]. Eight studies consider all cancers (solid and hematologic) [6, 30, 32, 33, 40, 42–44]; the rest consider only hematologic tumors [11, 39, 41, 45–48]. AKI is not always clearly defined and only eight studies use the RIFLE, AKIN, or KDIGO classifications [11, 30, 32, 39, 40, 44, 46, 47], usually only taking into account SCr and not urinary volume. It is highly probable that in some papers the need for RRT was used as definition of AKI [6, 40, 42, 45, 48], which creates a risk of underestimation of AKI incidence, at least in part explaining the variability in different reports. Furthermore, the incidence of AKI on admission was sometimes lower than the number of patients in whom RRT treatment was needed after admission, suggesting that AKI very frequently develops during the ICU stay [43, 45]. Many studies start from the oncologic viewpoint and consider AKI as one of the many determinants of outcome, not considering specific aspects related to AKI or a comparison with patients without AKI. Vice versa, studies concentrating on the AKI aspect rarely contain a comparison with subjects without cancer. The picture that arises is one of a population which is extremely vulnerable to AKI with up to 60 % of patients developing AKI [11, 39], and up to 40 % of them in need of RRT [43, 45]. Main causes associated with AKI are sepsis and septic shock [6, 39, 40, 42, 47], tumor lysis [11, 39, 43], and nephrotoxic agents [11, 39, 46].

**Fig. 1:**
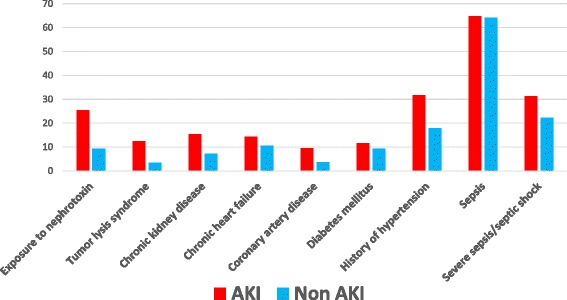
Factors associated with AKI in hemato-oncology patients. Adapted from Darmon et al. [39]. *p < 0.05. *AKI* acute kidney injury

Figure 1 illustrates the different major risk factors of AKI in 1009 critically ill cancer patients with (*n* = 671) and without (*n* = 338) AKI, taken from the recent paper by Darmon et al. [39]. Although many risk factors were present in both patient groups, severe sepsis, a history of hypertension, exposure to nephrotoxic agents, preexistent chronic kidney disease, and TLS were significantly more frequent in the AKI patients.

Length of stay in the ICU is, in general, longer for cancer patients with AKI than for those without AKI [11, 32], but in one study the stay was not longer than for noncancer patients with AKI [40]. Length of hospital stay, if mentioned, is the same in AKI patients with and without cancer [11, 40, 44]. In one study [11], AKI patients showed less complete remission of the malignancy (39.4 %) compared with patients without AKI (68.3 % remission). Long-term kidney function is rarely mentioned and appears not to recover in all cases; 23 % [47] or 12.9 % [39] of surviving hematologic patients needed RRT after ICU discharge. AKI patients with cancer have a higher mortality than those without cancer [6, 40], cancer patients with AKI have a higher mortality than those without AKI [11, 32, 39, 46], RRT is linked to an even higher mortality [39, 41–43], and mortality increases with severity of AKI [32, 46].

In future studies, potential attention points could be: the use of now generally accepted classification systems like RIFLE, AKIN, or preferably KDIGO; indications of RRT and length of ICU and hospital stay; comparisons of AKI vs no AKI in studies focusing on cancer, and of cancer vs no cancer in studies focusing on AKI; and long-term kidney function, and long-term survival and response to chemotherapy of cancer patients with AKI.

## Prevention of AKI in critically ill cancer patients

It is beyond the scope of this review to discuss the general prevention of AKI in critically ill patients. Recent comprehensive literature on this topic is available [17]. A specific cause of potentially dramatic AKI – acute TLS – requires the attention of all stakeholders; the hemato-oncologist, the nephrologist, and the intensivist.

## Tumor lysis syndrome

TLS is an oncologic emergency caused by the release of large amounts of potassium, phosphate, and nucleic acids into the systemic circulation secondary to massive tumor cell lysis and may lead to life-threatening AKI, arrhythmias, and neurologic complications. TLS typically occurs after the initiation of anticancer therapies, including cytotoxic drugs, biological agents, corticosteroids, hormones, and radiation therapy. TLS has been reported with virtually every type of tumor, but is typically associated with bulky, chemosensitive hematologic malignancies. The incidence of TLS in patients with solid malignancies may be higher than previously thought [49]. TLS may also arise spontaneously as a consequence of increased tumor cell lysis before any definitive antitumor therapy has been initiated (for recent reviews see [7, 50]). The 2004 Cairo and Bishop classification system for TLS made a distinction between laboratory TLS and clinical TLS, the latter defined as laboratory TLS accompanied by symptomatic complications, and was revised in 2010 [51]. However, the distinction between clinically relevant and laboratory defined TLS remains difficult and the Cairo and Bishop classification is therefore not uniformally accepted [50].

AKI in TLS is usually oliguric and has various mechanisms; most commonly, the deposition of uric acid crystals in distal tubules resulting in intraluminal obstruction is the main pathogenetic factor. Other possibilities are precipitation of calcium phosphate, renal infiltrates of tumor, tumor-related obstructive uropathy, drug-induced nephrotoxicity, preexisting renal impairment, dehydration, and sepsis.

### Risk assessment of TLS

General risk factors are common to most forms of AKI, like patient’s age and intravascular volume status, exposure to potentially nephrotoxic substances, and baseline kidney function. Certain intrinsic tumor-related risk factors include, among others, a high tumor cell proliferation rate, the degree of chemosensitivity of the malignancy, and the presence of a bulky disease >10 cm in diameter and/or a white blood cell count >50,000/μl. Certain biochemical parameters such as pretreatment serum lactate dehydrogenase (LDH) more than two times the upper limit of normal, pretreatment hyperuricemia (serum uric acid >7.5 mg/dl (>446 μmol/l)) or hyperphosphatemia and an abnormal SCr [50] are additional risk factors for TLS.

Subjects at intermediate and high risk of TLS should be monitored in a hospital setting and possibly in an ICU (especially individuals at high risk of TLS).

### Prevention of TLS

All patients who are at risk for the TLS should receive intravenous hydration to rapidly improve renal perfusion and glomerular filtration and to minimize acidosis and oliguria (an ominous sign). Hydration is realized by means of intravenous fluids (2500–3000 ml/m^2^ per day of preferably isotonic saline). Diuretics do not have a proven role in reducing the incidence or severity of TLS and their routine use is not recommended unless there are clinical signs or symptoms of volume overload [52]. The role of urinary alkalinization is unclear and controversial; no controlled human studies exist to inform the decision of whether to attempt alkalinization. Although alkalinization improves urate solubility, a high pH decreases the solubility of xanthine, hypoxanthine, and calcium phosphate, potentially increasing the likelihood of intratubular crystallization. If alkalinization is used in this setting, serum phosphorus should be followed carefully and should be stopped when hyperphosphatemia develops, to minimize the risk of acute phosphate nephropathy [53].

Allopurinol and rasburicase, a recombinant urate oxidase preparation, decrease the synthesis of uric acid in patients with rapidly growing tumors who are prone to uric acid nephropathy and TLS [50]. Allopurinol should be started at least 24–48 h prior to chemotherapy or radiation therapy to reduce the risk of uric acid nephropathy. Although allopurinol prevents the formation of uric acid, existing uric acid must still be excreted. The level of uric acid may take 2 days or more to decrease, a delay that allows urate nephropathy to develop. Despite treatment with allopurinol, xanthine may accumulate, resulting in xanthine nephropathy. Patients who do not tolerate oral medication can be given allopurinol intravenously. The recommended dose of allopurinol is up to 800 mg/day orally or 300 mg/m^2^, and up to 600 mg/day for the intravenous formulation [54]. The dose of allopurinol should be reduced in the presence of chronic kidney disease.

Febuxostat is a xanthine oxidase inhibitor that does not have the hypersensitivity profile of allopurinol and does not require dosing adjustments for reduced GFR. Like allopurinol, febuxostat is not expected to decrease the accumulation of xanthine or the risk for xanthine stone formation. Febuxostat may be a reasonable, albeit expensive, alternative to allopurinol in the prophylaxis of TLS in patients with decreased estimated GFR [52].

Rasburicase is a recombinant form of *Aspergillus*-derived urate oxidase and catalyzes the oxidation of uric acid to allantoin, which is 5–10 times more soluble than uric acid and is readily excreted. Rasburicase was approved by the US Food and Drug Administration (FDA) for children in 2002 and adults in 2009. The US FDA-recommended dosing guideline for rasburicase in pediatric patients is 0.15 mg/kg or 0.2 mg/kg administered once daily for a maximum of 5 days. Treatment beyond 5 days or for more than one course of therapy is not recommended. The first dose of rasburicase should be administered 4–24 hours before starting chemotherapy. Rasburicase is given as an intravenous infusion over 30 minutes and should not be given as a bolus infusion. Currently, in the adult population, the dose often utilized in practice is 0.2 mg/kg. Rasburicase is now a standard of care for patients at high risk of TLS despite continuing debate on whether its profound and rapid lowering of plasma uric acid levels translates into hard patient outcomes (e.g., AKI with a need for RRT or mortality). Two recent meta-analyses [55, 56] concluded that the mortality and cost-effectiveness benefits of this expensive drug remain to be proven. Several studies have reported higher rates of serious adverse reactions (pulmonary hemorrhage, respiratory failure, supraventricular arrhythmias, ischemic coronary artery disorders, and abdominal and gastrointestinal infections) with rasburicase compared with allopurinol [57].

The liberation of hydrogen peroxide induced by rasburicase can be devastating in patients with glucose 6-phosphate dehydrogenase (G6PD) deficiency, an abnormality affecting 400 million people worldwide, especially in regions with endemic malaria [58]. In these individuals the unchecked oxidative potential of hydrogen peroxide can lead to methemoglobinemia and hemolytic anemia [59]. Particularly in patients of African, Mediterranean, or Southeast Asian ancestry, testing for G6PD deficiency is warranted before the administration of potent oxidizing agents (e.g., rasburicase).

Acute phosphate AKI can also be part of TLS and it has been suggested that wide use of rasburicase and urine alkalinization has resulted in a paradigm shift towards acute phosphate nephropathy in TLS-induced AKI [60]. TLS patients may develop a first episode of AKI secondary to spontaneous TLS-induced acute urate nephropathy, treated with rasburicase and allopurinol, and a second episode due to chemotherapy-induced TLS with acute phosphate nephropathy.

### Established TLS

Several features of the preventive management of TLS remain the mainstay also for the treatment of established TLS. RRT should be considered for all critically ill patients with TLS having persistent metabolic abnormalities or renal failure in spite of fluid replacement. Between 40 and 71 % of patients suffering from TLS develop a need for RRT [61]. The selection of RRT in established TLS is briefly discussed in the next section.

## RRT modality in critically ill patients with cancer with AKI

Older studies on dialysis in patients with cancer were discussed extensively in a previous review [10]. As indicated in Table 1, various acute dialysis modalities are used in critically ill cancer patients. Continuous dialysis modalities are often preferred over intermittent therapies to start RRT in hypotensive oliguric patients who require vasopressors and large volumes of fluids. In an earlier study in our unit, continuous renal replacement therapy (CRRT) was initially used in 71.4 % vs 43.1 % of AKI patients with vs without hematological malignancies [9]. Many ICU centers start with CRRT and switch afterwards to intermittent hemodialysis (IHD) when the hemodynamic situation of the patient has stabilized.

A hybrid therapy called sustained low-efficiency dialysis (SLED) or extended dialysis (EDD) has emerged as an alternative to CRRT in the management of hemodynamically unstable patients with AKI [62]. Salahudeen et al. [63] retrospectively assessed 199 ICU cancer patients requiring RRT. Most patients (62 %) had hematological cancers, sepsis was present in 27 % of the cases, and 30-day mortality was 65 %. All patients received “continuous” SLED (sustained low-efficiency extended dialysis). Although 75 % of the patients were on vasopressors before dialysis initiation, satisfactory ultrafiltration with acceptable hemodynamic stability were achieved. This study was the first to describe the technical characteristics of SLED in patients with cancer in the ICU.

In a recent study with mainly solid cancer patients, IHD was the initial RRT modality in 108 (72.5 %) patients [64]. The other 27.5 % were transferred to IHD after 4.0 (2.0–7.0) days of CRRT. Thirty-eight (25.5 %) patients were transferred from IHD to CRRT due to hemodynamic instability. Fifty (34.1 %) of the patients received both IHD and CRRT modalities during their ICU stay. Overall, in these critically ill patients with cancer and AKI, IHD offered acceptable hemodynamic stability and provided adequate metabolic control.

It is the author’s opinion that irrespective of the etiology of AKI the choice of intermittent vs continuous RRT should be based on the experience of the ICU and nephrology team and the availability of therapies. When both therapies are available, the indication of CRRT or IHD is based on the patient’s neurologic, hemodynamic, and catabolic status. Ideally, the therapy should be tailored to the patient’s demands, which changes daily in the critically ill. It is now accepted that more than one therapy will be utilized for managing patients during the course of AKI. Transitions from CRRT to IHD are common and reflect the changing needs of patients during their AKI course. For instance, patients in the ICU may initially start on CRRT when they are hemodynamically unstable, transition to SLED–EDD when they improve, and leave the ICU on IHD. Whenever possible, all dialytic modalities should be utilized as indicated to best support patient needs through their course.

CRRT should be preferred, however, for patients with acute brain injury or other causes of increased intracranial pressure of generalized brain edema.

There are some special considerations in the selection of RRT in TLS. Given the potential ongoing liberation of substances from lysing cells in TLS, continuous therapies are often preferred. However, IHD with the attendant rapid clearance of potassium may be the modality of choice for life-threatening hyperkalemia; this can then be followed by a continuous therapy at high dialysate or replacement fluid flow rates (of 3–4 l/h) to avoid rebound hyperkalemia and provide ongoing dialytic therapy for TLS [65]. CRRT, such as continuous venovenous hemofiltration (CVVHF), continuous venovenous hemodialysis (CVVHD), or combination therapy, may be preferable in the setting of severe hyperphosphatemia. A high dialysate flow rate (3–4 l/h) may be necessary to adequately maintain clearance in the face of active liberation of cellular contents [65].

Because acute peritoneal dialysis achieves inadequate uric acid clearance, its routine use is not recommended in TLS.

In recent years there has been great interest in novel RRTs in myeloma cast nephropathy, the most frequent form of AKI in multiple myeloma (MM) patients. AKI is a frequent form of myeloma presentation and besides the common causes of AKI some particular causes must be considered in the extensive differential diagnosis, such as hypercalcemia, glomerular amyloid or nonamyloid deposits, cast nephropathy, and infrequent kidney infiltration by myeloma cells. Up to 90 % of cases of severe AKI in MM are secondary to myeloma cast nephropathy (for review see [14]).

Prompt institution of effective antimyeloma chemotherapy remains the mainstay for the treatment of patients with myeloma kidney, as response to chemotherapy is the most important prognostic factor. New MM agents which are well tolerated in patients with severe AKI (for instance, bortezomib) have dramatically improved the prognosis of MM patients with AKI at presentation, and recent studies report a renal recovery rate of 30–60 % with an overall survival of 21.5 months [66]. Effective suppression of the proliferative clone by chemotherapy usually requires several days to weeks following treatment initiation, and extracorporeal removal of free light chains (FLCs) using plasmapheresis or other dialytic techniques has been reported as an adjunct to remove pathogenic serum FLCs until the proliferative clone is controlled by chemotherapy (for review see [67]).

Randomized controlled trials (RCTs) that have evaluated the efficacy of plasma exchange as an adjunct to chemotherapy argue against the use of this procedure in the routine management of AKI associated with MM [67]. By contrast, recent clinical data suggest that using a dialyzer cartridge with pores of sufficient size to allow the removal of FLCs using a newly developed polyflux® high cut-off (HCO) 1100 protein-leaking dialyzer in conjunction with effective chemotherapy can achieve sustained reduction of serum FLC concentrations and may potentially improve cast nephropathy [66]. Yadav et al. [68] recently showed that an optimal approach, including new MM agents in patients with AKI requiring RRT, led to a 50 % rate of renal recovery. Encouraging was the observation that patients who actually recovered had a very good long-term prognosis characterized by an overall survival of 64 months. Moreover, more than 95 % of patients remained dialysis independent until death or end of follow-up. Serum FLC reduction at day 21 from baseline predicted the long-term GFR. Roughly half of the patients who became dialysis-free were dialyzed with either standard high-flux hemodialysis (HF-HD) or extended high cut-off hemodialysis (HCO-HD). Due to the small numbers in this study it is impossible to make any firm conclusion whether HCO-HD resulted in better outcomes compared with HF-HD [68].

Unfortunately, there is at present no firm evidence that HCO dialysis adds significant clinical benefits to chemotherapy alone [67]. Thus, until convincing evidence is available, the use of plasmapheresis and other extracorporeal FLC removal techniques should be limited to carefully selected patients within established research protocols. A number of RCTs are currently on their way: European Trial of Free Light Chain Removal (EULITE) (controlled clinical trials ISRCTN45967602); and Studies in Patients with Multiple Myeloma and Renal Failure due to cast Nephropathy (MYRE) (ClinicalTrials.gov NCT01208818).

## Increased risk of CKD

AKI also increases the risk of incident CKD or accelerates the progression of preexisting CKD (for recent review see [69]).

Among long-term survivors of HSCT, the average prevalence of CKD in children and adults, based on studies from 2007 onwards, was estimated to be 13 % [70].

A recent retrospective, longitudinal study of patients who survived more than 10 years after myeloablative allogeneic HSCT showed a cumulative increased incidence of CKD which reached 34 % at 10 years [71]. Patients who did not have AKI did not develop CKD and the adjusted hazard ratio increased with the severity of AKI (based on AKIN classification). Patients are more likely to develop CKD in the first year following HSCT (15 %) than in subsequent years [71]. The increased risk of CKD after AKI is particularly relevant in long-term pediatric cancer survivors [72]. The precise mechanism by which AKI accelerates CKD in humans is an area of ongoing active research with the understanding of the genesis of interstitial fibrosis as a central focus of study (for recent reviews see [69]).

Recognizing that CKD is an important risk factor for cardiovascular disease, progression to ESRD, infection, hospitalization, and death, it is clear that preventing its development by preventing AKI is also an important goal among cancer patients.

## Conclusions

Recent literature confirms that AKI requiring RRT is more common in ICU patients with cancer than in those without, and that ICU and hospital mortality rates are high in patients with cancer and AKI, especially when RRT is required. However, long-term survival is no longer exceptional even in multiple organ failure cancer patients requiring RRT. This improvement is probably due to better ICU admission policies and triage of patients who should receive RRT, new and more efficient anticancer treatments, and better supportive care overall during and after the ICU stay. Good collaboration and open and honest communication between intensivists, hemato-oncologists, and nephrologists is necessary in order to further improve the prognosis of these very complex patients and to guarantee that the degree of advanced life-supporting therapy remains proportional to their expected long-term prognosis and quality of life during their entire ICU stay.

## Abbreviations

AKI, acute kidney injury; AKIN, Acute Kidney Injury Network; CKD, chronic kidney disease; CRRT, continuous renal replacement therapy; CVVHD, continuous venovenous hemodialysis; CVVHF, continuous venovenous hemofiltration; CysC, Cystatin C; EDD, extended daily dialysis; GFR, glomerular filtration rates; HSCT, hematopoietic stem cell transplantation; IHD, intermittent hemodialysis; KDIGO, Kidney Diseases Improving Global Outcomes; MM, multiple myeloma; NICE, National Intensive Care Evaluation; RCT, randomized controlled trial; RIFLE, Risk, Injury, Failure, Loss, End stage renal disease; RRT, renal replacement therapy; SCr, serum creatinine; SLED, sustained low-efficiency dialysis; TLS, tumor lysis syndrome; UO, urine output
